# Supplementary material for: Chromothripsis during telomere crisis is independent of NHEJ, and consistent with a replicative origin
Source: Genome Res. 2019 May;29(5):737–49. doi: 10.1101/gr.240705.118 (PMC6499312; doi:10.1101/gr.240705.118)
Supplement: Supplemental Material [file supp_gr.240705.118_Supplemental_file_1.zip › contigs/annotated_contigs/DB89/contig.2.DB89_length_668_mean_cov_10.8532934132.docx]

**DB89_length_668_mean_cov_10.8532934132**

```
AATGTTGATGAGTATATAGAGCTACTAGAATTCACACATATTGTTAGTGGGAGTGTTAAATGATGCAACCATTTTGGAAAACAGCTGGA
 >chr22:28365631-28366086 - E=3e-257
TAGTTTCTTATAAAATTGAACATACACCCACTCTATCGCCCAATCAAATTCTATACCTAAGTATTCACTCAAGAGAAATGAAAAAAGAT

GTCCACAAAGAGACTCATCTAAGAATGTTCATAGTTGCCTTATCCATAGCAGCCACAACTGGAAAAAACAAAATGCCTATTATCAGGTA

AATGGATAAACAAATTTTGGTATAGTCGTACAGTGGAATACTCAACAACAAAAAGGAATGAACTGATATATCCACCAAGATGAGTGAAT

ATAAAACACTGTATGTTGAGCAAAATAAGTAAAAAACAAGAGTTCATACGTTTCTATTTATACAGAGTCCTAGAATAATCGAAACCTAC

TTACGA|TGA|AACTGATGAATTCACTTCAATTCCAACCTAATCAATTCTGAACTAGTGCCTTGAAGTCATTTAAATAGTAACTTCCTT
       >chrX:10604546-10604763 - E=8e-119
TGCACAAAATCTTAAGACCGTGGAGAGTGGAGGTCAAGAGAACACTAGTAAGGTGGCCTCTGACTTCTTGAGCTTTTGACATTCAGCCA

CCAGAGGCTGCTCTGAGACCATTATCCTCTTTTCACTCCGGGTCCAA
```
